# Perceptions of best practice, pain science and structure-focused education for rotator cuff-related shoulder pain: a content analysis of qualitative data from a randomised experiment

**DOI:** 10.1136/bmjopen-2025-107032

**Published:** 2026-02-06

**Authors:** Zixin Zhang, Giovanni E Ferreira, Ryan Muller, Fernando Sousa, Peter Malliaras, Courtney A West, Mary O’Keeffe, Chris Maher, Joshua Zadro

**Affiliations:** 1 Institute for Musculoskeletal Health, Sydney Local Health District, Camperdown, New South Wales, Australia; 2 School of Public Health, Faculty of Medicine and Health, The University of Sydney Institute for Musculoskeletal Health, Camperdown, New South Wales, Australia; 3 VA Connecticut Healthcare System, West Haven, Connecticut, USA; 4 Monash University Department of Physiotherapy, Frankston, Victoria, Australia; 5 UCD School of Public Health, Physiotherapy and Sports Science, University College Dublin, Dublin, Ireland

**Keywords:** Shoulder, Health Education, QUALITATIVE RESEARCH

## Abstract

**Objectives:**

To explore how people perceive different forms of education for rotator cuff-related shoulder pain in terms of words or feelings evoked by the education and treatments they feel are needed.

**Setting:**

We performed a content analysis of qualitative data collected in a randomised experiment.

**Participants:**

2237 participants with rotator cuff-related shoulder pain were randomly assigned to receive three forms of education: *best practice education*, *best practice education plus pain science messages* and *structure-focused education*.

**Primary and secondary outcomes:**

After receiving the education, participants answered two questions regarding (1) words or feelings evoked by the education and (2) treatments they felt were needed.

**Results:**

2232 responses for each question were analysed (99.7% response rate). Participants who received *best practice education* more frequently expressed feelings of unhappiness/frustration. The addition of *pain science messages* to *best practice education* resulted in slightly more emotional responses and a greater sense of being validated or cared for. In contrast, participants who received *structure-focused education* more frequently expressed trust in the clinician’s expertise and the need for medication, activity modification, rest, diagnostic imaging, injections and surgery. These participants also less frequently considered exercise as a viable treatment option.

**Conclusion:**

Participants with rotator cuff-related shoulder pain expressed generally similar emotional responses across groups, with small differences in treatment preferences favouring self-management in the *best practice education* groups compared with those who received *structure-focused education*. Those in the *best practice education* also less frequently reported needing potentially unnecessary treatments (eg, imaging, injections and surgery).

**Trial registration number:**

Australia New Zealand Clinical Trials Registry (ACTRN12623000197639).

**STRENGTHS AND LIMITATIONS OF THIS STUDY**

- This study used a large, international sample of people with rotator cuff-related shoulder pain.
- This study was conducted using high-quality methods, including randomisation and allocation concealment, which reduce the risk of bias.
- The content analysis was methodologically rigorous, with the coding framework adapted from frameworks used in prior research.
- A limitation of this study was that it was conducted online, which may limit the generalisability of the findings to real-world clinical settings.
- The timing of data collection prevented assessment of the long-term effects of education, as all responses were collected immediately after the interventions.

## Introduction

 Rotator cuff-related shoulder pain is the most common cause of shoulder pain,^1^ with an estimated prevalence of 85% in the general population. It is an umbrella term encompassing a range of conditions involving the rotator cuff (eg, tendinopathy, tear and calcific tendinitis) and surrounding structures (eg, subacromial bursitis). This term excludes other distinct causes of shoulder pain, such as adhesive capsulitis, glenohumeral osteoarthritis, glenohumeral instability, fractures or dislocations or cancerous tumours in the shoulder.^2^ Given the broad and overlapping nature of this diagnosis, the labels used to describe rotator cuff-related shoulder pain vary considerably. Common labels include ‘bursitis’, ‘rotator cuff tear’ and ‘subacromial impingement syndrome’, each reflecting differing clinical interpretations of the underlying pathology.^3^

Advice and education are recommended in many clinical guidelines for the management of musculoskeletal conditions.^4^,^6^ While advice typically refers to recommending a course of action, education involves imparting knowledge. In line with prior literature,^7^ the term ‘education’ in this paper encompasses both ‘advice’ and ‘education’. For conditions such as back pain and neck pain, most guidelines recommend educational approaches that encourage people to stay active,^8 9^ provide reassurance about prognosis^8^ and advise against prolonged rest.^8 9^ In contrast, most guidelines for rotator cuff-related shoulder pain offer limited direction on the type of education patients should receive. A recent overview of nine clinical guidelines for rotator cuff-related shoulder pain found that only one recommended education focused on resuming daily activities and understanding the benefits and harms of treatment options,^10^ while three recommended education on medication use. Notably, no guideline provided specific recommendations regarding the content or delivery of patient education.^10^

Given the lack of clear recommendations for providing education in the management of rotator cuff-related shoulder pain, we conducted an online randomised experiment (n=2237) to examine the effects of different educational approaches. In this online experiment, *best practice education* emphasised that most shoulder pain is not serious and is not a reliable indicator of tissue damage, while encouraging simple self-management strategies. *Structure-focused education* emphasised structural changes as being responsible for pain and should be targeted with treatment.^7^ *Best practice education* led to greater reassurance and intentions to try simple, low-cost management strategies (eg, exercise, heat, cold, simple pain medications and activity modification) compared with *structure-focused education*.^7^ Adding *pain science messages* to *best practice education*, which aims to help patients understand the complex nature of pain, had a similar effect as *best practice education* alone, but led to further increases in reassurance.^7^

As part of this online experiment,^7^ we collected qualitative data to better understand why reassurance and treatment preferences varied according to the type of education participants received. The aim of this study was to explore how people with rotator cuff-related shoulder pain perceived *best practice education* (with or without pain science *messages*) and *structure-focused education* in terms of (1) the words or feelings evoked by the educational content and (2) the treatments they believed were necessary for managing their condition.

## Materials and methods

### Study design

We conducted a content analysis of qualitative data collected in a three-arm, online randomised controlled experiment involving participants with rotator cuff-related shoulder pain.^7^

### Participants and recruitment

Participants with rotator cuff-related shoulder pain were recruited through Qualtrics (www.qualtrics.com) from May to June 2023 from Australia, New Zealand, Canada, United Kingdom and USA. Qualtrics uses existing panels of individuals who have previously agreed to complete online surveys (‘market research panels’) and have more than 20 panel providers globally to recruit research participants across various fields (eg, travel, finance, healthcare, technology and retail). Participation is voluntary, and participants must be 18 years or older and able to read and type in English. Qualtrics uses random sampling and provides incentives for participation (eg, cash, airline miles and gift cards). Qualtrics checks IP addresses and uses digital fingerprinting software to reduce duplication and ensure valid responses. More details on Qualtrics’ sampling and recruitment procedures can be found elsewhere.^11^

### Data collection

Potential participants recruited via Qualtrics were asked to provide informed consent by checking a box, followed by completing four screening questions to determine eligibility. These questions ensured inclusion of participants who (a) self-identified as currently experiencing shoulder pain, (b) rated their shoulder pain in the past week as ≥1 on a 0–10 scale, (c) reported pain in the anterolateral shoulder and upper arm and (d) had not been diagnosed by a health professional with adhesive capsulitis, glenohumeral osteoarthritis, glenohumeral instability, a shoulder fracture or dislocation or cancer or infection in the shoulder. We did not capture further information about symptom characteristics to reduce the burden of these screening questions on participants and since our screening criteria approximate a diagnosis of rotator cuff-related shoulder pain.^12^

To minimise the risk of ineligible participants falsely reporting in order to receive incentives, the first screening question listed multiple pain locations (eg, whole body and shoulder). Participants could select a maximum of three pain locations, and only those who selected shoulder pain were permitted to complete the survey. The screening questions and survey can be found in online supplemental file 1 and elsewhere.^7^

Eligible participants then completed a series of questions regarding demographics (eg, sex, age and educational attainment), healthcare utilisation (eg, history of diagnostic imaging, physiotherapy and injections) and their shoulder condition. Further details on data collection are provided elsewhere.^7^

Participants were randomised in a 1:1:1 ratio using Qualtrics survey software into one of three groups: (1) *best practice education*, (2) *best practice education plus pain science messages* or (3) *structure-focused education*.

The three forms of education were as follows:

- *Best practice education (3 min, 52 s*), which highlights ideas that most shoulder pain is not serious or a good indicator of tissue damage and recommends simple self-management strategies (eg, heat, cold, simple pain medicines, activity modification and exercise).
- *Best practice education plus pain science messages (5 min, 53 s*) which attempt to help patients understand the complex nature of pain (eg, role of brain and nervous system).
- *Structure-focused education (2 min, 12 s*) highlighting that structural changes are responsible for pain and should be targeted with treatment.

All rationale for choosing these specific forms of education is described elsewhere.^7^

All education was delivered via pre-recorded videos (ranging from 2 to 6 min) by the same professor and physiotherapist (PM). Each video was accompanied by a written transcript, which was accessible to participants. The length of each intervention video was determined by the amount of information provided. For example, the combined *best practice education with pain science messages* video was a longer video as more information was required than the best practice education video alone. The intervention video was displayed immediately after randomisation, on the same Qualtrics page. Participants were required to remain on the page for the full duration of the video, and they were able to rewatch it if desired.

Outcome data were collected immediately following the video intervention. In this paper, we focused on participants’ free-text responses to two open-ended questions:

- If your health professional gave you this information, how would it make you feel?
- If your health professional gave you this information, what treatments (if any) do you think you would need?

### Data analysis

Content analysis^13^ was used to analyse responses to the free-text questions, allowing us to report the frequency of codes present in participants’ answers. To complete the content analysis, a physiotherapy researcher (JZ) and three Master of Public Health students (YH, CC, AY) familiarised themselves with the responses and reviewed a coding framework from a prior content analysis^14^ that examined how people interpret brief educational messages about shoulder pain using similar free-text questions. This framework served as a foundation for developing the coding framework used in the current study.

Initial coding was completed in pairs by the Master of Public Health students using an inductive approach, ensuring each response was double-coded. Discrepancies were resolved by discussion or by consulting with a different researcher (JZ). An additional PhD student with a background in sports medicine (ZZ) independently reviewed the responses to triple code the responses and to identify discrepancies in coding against the framework. ZZ had some experience in coding content analyses prior to this study but was supervised by JZ who has extensive experience in content analysis.^15^,^17^ All responses were coded independently, and all disagreements were resolved through further consultation with JZ. All coders were blinded to group allocation.

The coders’ expertise mostly relates to conservative management of rotator cuff-related shoulder pain, which may have influenced the lens by which the data were interpreted. However, the use of double coding and discussion between coders strengthened the trustworthiness of the analysis, and the coding framework adapted from previous studies^15 17 18^ likely reduced any subjective interpretations related to the coders’ background and expertise.

Each response was assigned as many codes as appropriate, with a maximum of six codes applied to a single response. The final coding frameworks are provided in online supplemental file 2. Frequency counts and percentages of each code were calculated using Microsoft Excel.

### Patient and public involvement

Patients and members of the public were not involved in the design, data collection, analysis, interpretation, validation or dissemination of the results.

## Results

### Sample characteristics and level of agreement

**Figure 1:**
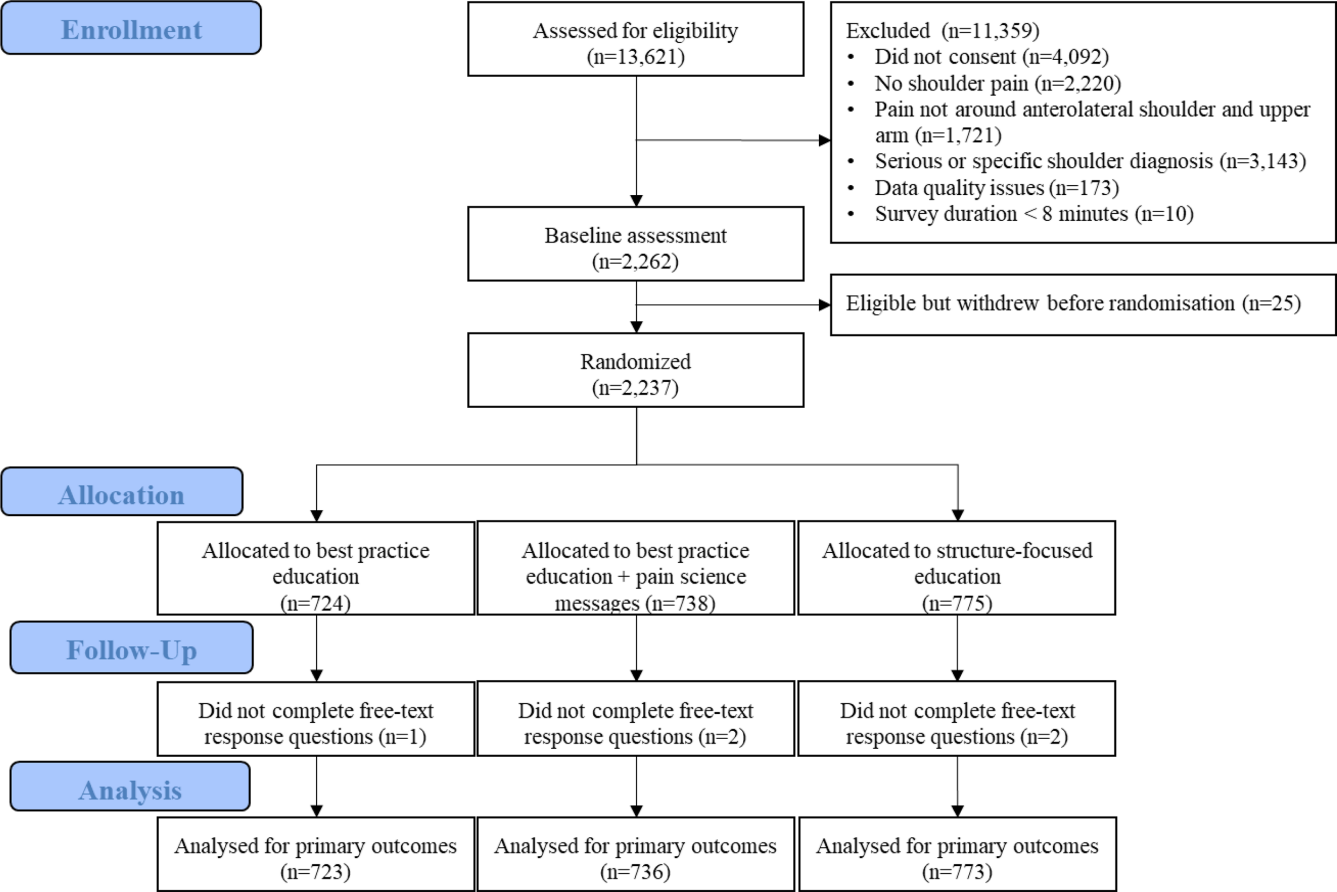
Flow diagram.

In our online experiment, 2237 eligible participants were randomised to receive one of the three types of education (figure 1). Five participants (0.3%) did not respond to the two open-ended questions, leaving 2232 (99.7%) responses to each question for this analysis. Participant characteristics were similar between the three groups (table 1).

**Table 1:** Participant characteristics

Demographics	Best practice education (n=724)	Best practice education+pain science messages (n=738)	Structure-focused education (n=775)
Age (years), mean (SD)	46.1 (16.1)	45.9 (16.1)	46.3 (16.4)
Female, n (%)	450 (62%)	450 (61%)	484 (63%)
Country, n (%)			
Australia	157 (22%)	165 (22%)	180 (23%)
Canada	162 (22%)	175 (24%)	169 (22%)
New Zealand	146 (20%)	122 (17%)	152 (20%)
United Kingdom	100 (14%)	123 (17%)	120 (16%)
USA	159 (22%)	153 (21%)	154 (20%)
Education, n (%)			
Up to high school (not completed)	57 (8%)	47 (6%)	54 (7%)
High school (completed)	194 (27%)	179 (24%)	221 (29%)
Non-university tertiary education	129 (18%)	141 (19%)	144 (19%)
University	344 (48%)	371 (50%)	356 (46%)
Anxiety (0–10, higher scores indicate greater anxiety), mean (SD)	4.8 (3.0)	4.7 (2.9)	4.6 (3.0)
Depression (0–10, higher scores indicate greater depression), mean (SD)	4.2 (3.1)	4.1 (3.2)	4.1 (3.2)
Healthcare use			
Previous imaging for shoulder pain, n (%)	149 (21%)	141 (20%)	171 (23%)
Previous physiotherapy for shoulder pain, n (%)	173 (24%)	166 (23%)	173 (22%)
Previous injection for shoulder pain, n (%)	90 (12%)	85 (12%)	94 (12%)
Previous surgery for shoulder pain, n (%)	43 (6%)	41 (6%)	48 (6%)
Previous sick leave for shoulder pain, n (%)	126 (17%)	137 (19%)	144 (19%)
Previous education for shoulder pain, n (%)	260 (36%)	242 (33%)	282 (36%)
Shoulder symptoms			
Shoulder pain over the last week (0–10, NRS), mean (SD)	5.3 (2.4)	5.2 (2.4)	5.1 (2.4)
Duration of shoulder pain, n (%)			
<1 week	186 (26%)	191 (26%)	196 (25%)
1 week to 3 months	200 (28%)	232 (31%)	216 (28%)
4 to 12 months	126 (17%)	123 (17%)	139 (18%)
>12 months	212 (29%)	192 (26%)	224 (29%)
Kinesiophobia (0–10, higher scores indicate greater fear of activity), mean (SD)	4.0 (3.0)	4.1 (3.1)	4.0 (3.0)
Total SPADI (0–100), mean (SD)	41.1 (23.2)	40.9 (23.8)	39.7 (23.6)
Pain subscore (0–100)	47.0 (23.3)	46.4 (23.6)	45.5 (23.6)
Disability subscore (0–100)	35.3 (25.2)	35.3 (26.0)	33.8 (25.5)

NRS, Numerical Rating Scale; SPADI, Shoulder Pain and Disability Index.

#### Question 1: ‘If your health professional gave you this information, how would it make you feel?’

Our coding framework identified 28 codes (table 2; examples of participants’ free-text responses provided in online supplemental file 3). The most commonly reported codes across all three groups were feeling positive about the education (31%–35%), reassurance (27%–31%), empowered (9%–11%), no impact on thoughts or feelings (6%–7%), willingness to follow the education (5%–6%) and trust in the healthcare professional’s expertise (4%–6%).

**Table 2:** Codes for words or feelings by message of education

Ranking	Total (n=2232)	Best practice education (n=723)	Best practice education+pain science messages (n=736)	Structure-focused education (n=773)
1	Positive about the education 730 (33%)	Positive about the education 227 (31%)	Positive about the education 261 (35%)	Positive about the education 242 (31%)
2	Reassurance 631 (28%)	Reassurance 224 (31%)	Reassurance 197 (27%)	Reassurance 210 (27%)
3	Empowered 219 (10%)	Empowered 72 (10%)	Empowered 63 (9%)	Empowered 84 (11%)
4	No impact on thoughts and/or feelings 132 (6%)	No impact on thoughts and/or feelings 41 (6%)	No impact on thoughts and/or feelings 44 (6%)	No impact on thoughts and/or feelings 47 (6%)
5	Willing to follow the education 121 (5%)	Willing to follow the education 38 (5%)	Willing to follow the education 36 (5%)	Willing to follow the education 47 (6%)
6	Trust in expertise 104 (5%)	Trust in expertise 28 (4%)	Trust in expertise 33 (4%)	Trust in expertise 43 (6%)
7	Good prognosis 60 (3%)	Unhappy/frustrated 24 (3%)	Good prognosis 22 (3%)	Need more information or options 24 (3%)
8	Treatment/investigation 54 (2%)	Need more information or options 19 (3%)	Validated or cared for 21 (3%)	Treatment/investigation 22 (3%)
9	Need more information or options 50 (2%)	Good prognosis 17 (2%)	Treatment/investigation 15 (2%)	Good prognosis 21 (3%)
10	Validated or cared for 46 (2%)	Treatment/investigation 17 (2%)	Minor issue 14 (2%)	Minor issue 18 (2%)
11	Uncertainty 45 (2%)	Uncertainty 16 (2%)	Uncertainty 14 (2%)	Validated or cared for 15 (2%)
12	Minor issue 44 (2%)	Have received similar advice before 16 (2%)	Negative about the advice 12 (2%)	Uncertainty 15 (2%)
13	Unhappy/frustrated 44 (2%)	Negative about the advice 15 (2%)	Unhappy/frustrated 11 (1%)	Negative about the advice 13 (2%)
14	Negative about the advice 40 (2%)	Minor issue 12 (2%)	Have received similar advice before 11 (1%)	Psychological distress 12 (2%)
15	Have received similar advice before 37 (2%)	Validated or cared for 10 (1%)	Need more information or options 7 (1%)	Have received similar advice before 10 (1%)
16	Psychological distress 22 (1%)	Feels dismissed 9 (1%)	Psychological distress 6 (1%)	Unhappy/frustrated 9 (1%)
17	Feels dismissed 19 (1%)	Avoid treatment/investigation 8 (1%)	Feels dismissed 4 (<1%)	Avoid treatment/investigation 8 (1%)
18	Avoid treatment/investigation 19 (1%)	Psychological distress 4 (1%)	Attention to pain 4 (<1%)	Feels dismissed 6 (1%)
19	Attention to pain 13 (1%)	Attention to pain 4 (1%)	Contrasting experience 4 (<1%)	Attention to pain 5 (1%)
20	Activity restriction 7 (<1%)	Second opinion 4 (1%)	Avoid treatment/investigation 3 (<1%)	Activity restriction 5 (1%)
21	Second opinion 7 (<1%)	Mechanism of injury 3 (<1%)	Second opinion 3 (<1%)	Mechanism of injury 1 (<1%)
22	Mechanism of injury 5 (<1%)	Contrasting experience 1 (<1%)	Activity restriction 2 (<1%)	Ageing 1 (<1%)
23	Contrasting experience 5 (<1%)	Ageing 1 (<1%)	Mechanism of injury 1 (<1%)	Poor prognosis 1 (<1%)
24	Ageing 3 (<1%)	Negative about the tone or presentation of the advice 1 (<1%)	Ageing 1 (<1%)	Contrasting experience 0 (0%)
25	Poor prognosis 2 (<1%)	Tissue damage or dysfunction 1 (<1%)	Poor prognosis 1 (<1%)	Second opinion 0 (0%)
26	Negative about the tone or presentation of the advice 1 (0%)	Poor prognosis 0 (0%)	Negative about the tone or presentation of the advice 0 (0%)	Negative about the tone or presentation of the advice 0 (0%)
27	Tissue damage or dysfunction 1 (0%)	Activity restriction 0 (0%)	Tissue damage or dysfunction 0 (0%)	Tissue damage or dysfunction 0 (0%)
28	Serious issue 0 (0%)	Serious issue 0 (0%)	Serious issue 0 (0%)	Serious issue 0 (0%)
29	Negative about an injection 0 (0%)	Negative about an injection 0 (0%)	Negative about an injection 0 (0%)	Negative about an injection 0 (0%)
30	Irrelevant response 149 (7%)	Irrelevant response 44 (6%)	Irrelevant response 55 (7%)	Irrelevant response 50 (6%)

Overall, emotional responses were largely similar across education types. However, there were some small differences observed. Participants who received *best practice education* more frequently expressed feelings of unhappiness/frustration (3%) versus (1% and 1%) compared with those receiving *best practice education plus pain science messages* (1%) and *structure-focused education* (1%). Those in the *best practice education plus pain science messages* group more frequently expressed positive feelings about the education (35% vs 31% in the other two groups) and feelings of being validated or cared for (3% vs 1% for *best practice education* and 2% for *structure-focused education*). Participants in the *structure-focused education* group marginally more often expressed trust in the health professional’s expertise (6% vs 4% in both other groups).

#### Question 2: ‘If your health professional gave you this information, what treatment(s) do you think you would need?’

A total of 40 codes were identified in the coding framework for this question (table 3). The most frequently mentioned perceived treatment needs across all three groups were exercise (11%–27%), wait and see (18%–21%), medication (13%–21%) and physiotherapy (11%–13%).

**Table 3:** Treatment codes by message of education

Ranking	Total (n=2232)	Best practice education (n=723)	Best practice education+pain science messages (n=736)	Structure-focused education (n=773)
1	Exercise 463 (21%)	Exercise 177 (24%)	Exercise 198 (27%)	Medication 159 (21%)
2	Wait and see 453 (20%)	Wait and see 153 (21%)	Wait and see 158 (21%)	Wait and see 142 (18%)
3	Medication 377 (17%)	Medication 120 (17%)	Medication 98 (13%)	Unsure 109 (14%)
4	Physiotherapy 266 (12%)	Physiotherapy 92 (13%)	Physiotherapy 80 (11%)	Physiotherapy 94 (12%)
5	Unsure 265 (12%)	Unsure 80 (11%)	Unsure 76 (10%)	Exercise 88 (11%)
6	Heat 152 (7%)	Heat 73 (10%)	Heat 61 (8%)	Rest 48 (6%)
7	Cold 129 (6%)	Cold 66 (9%)	Cold 47 (6%)	Diagnostic imaging 48 (6%)
8	Rest 101 (5%)	Light exercise 28 (4%)	Light exercise 44 (6%)	Injection 42 (5%)
9	Light exercise 95 (4%)	Rest 26 (4%)	Rest 27 (4%)	Surgery 41 (5%)
10	Diagnostic imaging 86 (4%)	Surgery 21 (3%)	Massage 21 (3%)	Follow the advice provided 26 (3%)
11	Surgery 73 (3%)	Normal movements 19 (3%)	Diagnostic imaging 20 (3%)	Activity modification 24 (3%)
12	Follow the advice provided 60 (3%)	Diagnostic imaging 18 (2%)	Follow the advice provided 18 (2%)	Light exercise 23 (3%)
13	Injection 58 (3%)	Follow the advice provided 16 (2%)	Normal movements 18 (2%)	Massage 19 (2%)
14	Massage 56 (3%)	Massage 16 (2%)	Treatment unspecified 12 (2%)	Heat 18 (2%)
15	Activity modification 47 (2%)	Activity modification 16 (2%)	Surgery 11 (1%)	Cold 16 (2%)
16	Normal movements 44 (2%)	Compression 13 (2%)	Education/advice 10 (1%)	Topical treatments 13 (2%)
17	Treatment unspecified 31 (1%)	Treatment unspecified 10 (1%)	Doctor 8 (1%)	Doctor 11 (1%)
18	Topical treatments 28 (1%)	Injection 9 (1%)	Activity modification 7 (1%)	Treatment unspecified 9 (1%)
19	Doctor 25 (1%)	Topical treatments 9 (1%)	Injection 7 (1%)	Normal movements 7 (1%)
20	Compression 25 (1%)	Doctor 6 (1%)	Topical treatments 6 (1%)	Education/advice 7 (1%)
21	Education/advice 21 (1%)	Education/advice 4 (1%)	Compression 6 (1%)	Compression 6 (1%)
22	Therapy 11 (<1%)	Therapy 4 (1%)	Therapy 3 (<1%)	Specialist 6 (1%)
23	Natural or unknown therapies 10 (<1%)	Natural or unknown therapies 4 (1%)	Follow-up appointments 3 (<1%)	Therapy 4 (1%)
24	Specialist 10 (<1%)	Ergonomics/posture 3 (<1%)	Manipulation 3 (<1%)	Natural or unknown therapies 4 (1%)
25	Plan 9 (<1%)	Plan 3 (<1%)	Specialist 2 (<1%)	Plan 4 (1%)
26	Ergonomics/posture 8 (<1%)	Follow-up appointments 2 (<1%)	Plan 2 (<1%)	Ergonomics/posture 3 (<1%)
27	Follow-up appointments 6 (<1%)	Specialist 2 (<1%)	Ergonomics/posture 2 (<1%)	Acupuncture 2 (<1%)
28	Acupuncture 5 (<1%)	Second opinion 2 (<1%)	Acupuncture 2 (<1%)	Chiropractor 2 (<1%)
29	Chiropractor 4 (<1%)	Acupuncture 1 (<1%)	Natural or unknown therapies 2 (<1%)	Follow-up appointments 1 (<1%)
30	Manipulation 4 (<1%)	Chiropractor 1 (<1%)	Emergency department/hospital 2 (<1%)	Manipulation 1 (<1%)
31	Diet 3 (<1%)	Diet 1 (<1%)	Chiropractor 1 (<1%)	Diet 1 (<1%)
32	Second opinion 3 (<1%)	Time off work 1 (<1%)	Diet 1 (<1%)	Second opinion 1 (<1%)
33	Time off work 3 (<1%)	Elevation 1 (<1%)	Time off work 1 (<1%)	Time off work 1 (<1%)
34	Elevation 2 (<1%)	Social support 1 (<1%)	Taping/bracing 1 (<1%)	Elevation 1 (<1%)
35	Social support 2 (<1%)	Taping/bracing 1 (<1%)	Psychological therapies 1 (<1%)	Social support 1 (<1%)
36	Taping/bracing 2 (<1%)	Manipulation 0 (0%)	Good mattress or pillows 1 (<1%)	Taping/bracing 0 (0%)
37	Emergency department/hospital 2 (<1%)	Emergency department/hospital 0 (0%)	Elevation 0 (0%)	Emergency department/hospital 0 (0%)
38	Psychological therapies 1 (0%)	Psychological therapies 0 (0%)	Social support 0 (0%)	Psychological therapies 0 (0%)
39	Good mattress or pillows 1 (0%)	Good mattress or pillows 0 (0%)	Second opinion 0 (0%)	Good mattress or pillows 0 (0%)
40	Irrelevant response 124 (6%)	Irrelevant response 34 (5%)	Irrelevant response 46 (6%)	Irrelevant response 44 (6%)

Perceived treatment needs were largely similar between the *best practice* and *best practice plus pain science* groups. However, notable differences emerged when compared with the *structure-focused education* group. Participants in the *structure-focused education* group more frequently believed they needed medication (21% vs 17% and 13% in the other groups), rest (6% vs 4% in both other groups), diagnostic imaging (6% vs 2% and 3% in the other groups), injections (5% vs 1% and 1%), surgery (5% vs 3% and 1%) and activity modification (3% vs 2% and 1%). They also less frequently considered exercise as a treatment option (11% vs 24% and 27% for *best practice education* and *best practice plus pain science messages*, respectively).

## Discussion

### Summary of key findings

The clearest differences in perceptions about different education types emerged in participants’ perceptions of which treatments were needed based on the information they received. Participants who received *structure-focused education*—which emphasises structural changes as the primary source of shoulder pain requiring targeted treatment—more frequently believed that they needed medication, rest, activity modification, diagnostic imaging (eg, X-ray, MRI and ultrasound), injections and surgery. They also less frequently considered exercise as a viable treatment option. In contrast, participants who received *best practice education*, which emphasises that most shoulder pain is not serious and encourages simple self-management strategies, less frequently expressed the need for those interventions. While emotional responses were broadly similar across education types, some small differences were observed. Participants who received *best practice education* more frequently expressed feelings of unhappiness/frustration compared with *best practice education plus pain science messages* and *structure-focused education*. The addition of pain *science* to *best practice education* also resulted in slightly more positive feelings about the advice and feelings of being validated or cared for. Participants who received *structure-focused education* more frequently expressed trust in the expertise compared with other educations.

These findings offer insights into how different educational approaches influence patient beliefs and expectations and may help inform future clinical guideline recommendations on the content and framing of education for people with rotator cuff-related shoulder pain.

### Strengths and weaknesses of this study

Key strengths of this study include the recruitment of a large, international sample of people with rotator cuff-related shoulder pain. Our sample appears broadly representative of people presenting with this condition in primary care, based on demographics and symptom profiles reported in prior studies.^119^,^21^ Our study used educational content from reputable sources, including the UK National Health Service, American Academy of Orthopaedic Surgeons Web page and a large clinical trial published in The Lancet.^22^ This enhances the relevance and real-world applicability of the findings. This study was part of a randomised experiment conducted using high-quality methods including randomisation and allocation concealment which reduce the risk of bias. The content analysis was also methodologically rigorous: the coding framework was adapted from frameworks used in prior research,^14 17 18^ and all responses were triple-coded to enhance reliability, and coding was conducted blinded to group allocation to minimise bias.

The main limitation of this study was that it was conducted online, which may limit the generalisability of the findings to real-world clinical settings. All educational materials were delivered via written text and prerecorded video, without any real-time interaction between participants and a health professional. In a clinical consultation, participants may receive more personalised information and have the opportunity to ask questions, potentially enhancing comprehension and engagement.

Additionally, the educational content in this study was delivered by a physiotherapist. It is possible that participants may have responded differently if the same information had been presented by other health professionals, such as general practitioners or surgeons, who may be perceived differently in terms of authority or expertise.^23^

Although we asked participants whether they had previously received education from a health professional, we did not explore what types of education participants had received. Repeated exposure to similar education may have had an impact on group differences in perceptions, as participants who previously received similar information may have had more time to reflect on how this education makes them feel and what treatments they feel are needed.

Another limitation was how we identified participants with rotator cuff-related shoulder pain. Our screening questions included participants if they self-identified as currently having shoulder pain with pain over the past week ≥1 on a scale of 0–10, had shoulder pain around the anterolateral part of the shoulder and upper arm and reported that they had not been diagnosed by a health professional with other specific or serious shoulder pathologies. These screening questions may be prone to recall bias if participants did not recall the specific or serious diagnosis they previously received from a health professional and may have captured some people with a specific diagnosis if they had not been formally diagnosed by a health professional. These inclusion criteria also likely captured some mild cases that may not reflect a clinical or care-seeking population. However, we consider this a minimal limitation because the mean pain scores in our sample are largely similar to other trials of people with shoulder pain and are therefore likely to be representative of the broader literature.^124^,^27^

Given the duration of education varied between groups, we cannot discount that the amount of information may have influenced the results. In addition, most responses were short (see online supplemental file 3), which may have limited the depth of the data we found.

Finally, all responses were collected immediately after the educational interventions, meaning we were unable to assess whether the effects of the education—such as treatment intentions or emotions—persisted over time.

### Meaning of the study

Our content analysis helps explain the quantitative findings from our online experiment.^7^ Most notably, the type of educational framing strongly influenced what participants thought they would need in terms of treatment. When pain was presented as primarily structural, participants more frequently endorsed diagnostic imaging (X-ray, MRI, ultrasound), injections, surgery, medication, rest and activity modification. They also less frequently considered exercise or other low-cost self-management options.

Conversely, *best practice education*—with or without *pain science messages*—prompted greater endorsement of simple, self-management strategies such as exercise, hot and cold application. These patterns mirror the trial’s quantitative results, where *structure-focused education* increased intentions to pursue unnecessary imaging and treatments (surgery), whereas *best practice education* bolstered intentions to self-manage.^7^ Together, the quantitative–qualitative convergence may highlight how a structural explanation for pain can nudge patients toward unnecessary and/or potentially harmful care, whereas best practice messages may encourage guideline-concordant care. These insights can inform future guideline recommendations and clinician–patient communication strategies for rotator cuff-related shoulder pain.

Emotional responses were broadly positive across groups, but there were some findings from this study that did not align with the quantitative findings of our online experiment.^7^ Our qualitative findings suggest that both *best practice education* (with or without *pain science messages*) and *structure-focused education* elicited similar positive feelings among people with rotator cuff-related shoulder pain, including feelings of reassurance. This contrasts with the quantitative findings, which showed that *best practice education* (with or without *pain science messages*) increased reassurance that no serious condition is causing pain and continuing with daily activities is safe.^7^ One possible explanation is that the reassurance outcomes in the quantitative results were specific to serious pathology and maintaining activity, whereas in this qualitative study, participants may have interpreted reassurance more broadly. *Best practice education* (with or without *pain science messages*) explicitly addressed serious pathology and maintaining activity, whereas *structure-focused education* did not. Therefore, not coding reassurance specific to these domains may explain why there were no differences in feelings of reassurance in this study.

### Comparison to the existing literature

Although this is the first study to examine how people perceive *best practice education* (with or without *pain science messages*) and *structure-focused education* for rotator cuff-related shoulder pain, our findings align with a previous qualitative study which found education emphasising structural changes can increase perceived need for unnecessary treatments (eg, imaging, surgery and injections) for shoulder pain.^14^ Our results also align with another qualitative study that explored the effects of guideline-based advice with or without pain science or ergonomics *messages* for people with acute low back pain.^17^ The study found that all three types of education generated similar positive feelings, reassurance and expectations among participants with acute low back pain.^17^ One possible explanation is that both our experiment and the acute low back pain trial used a more comprehensive and practical educational strategy—encompassing both recommendations for a course of action and imparting knowledge—delivered to participants with lived pain experiences. In contrast, other qualitative studies on shoulder pain^14^ or non-specific low back pain,^15^ which found significant differences between educational strategies, asked participants to respond to vignettes describing a hypothetical patient rather than drawing on their own pain experiences.

Our study found that adding *pain science messages* (which aim to help patients understand the complex nature of pain) to *best practice education* can encourage participants to try self-management strategies for rotator cuff-related shoulder pain. This type of education is also recommended by current clinical practice guidelines for low back pain^4 5^ and neck pain.^6^ For example, guidelines for low back pain typically recommend that education should provide information about the condition, address patients’ concerns and expectations about management and offer reassurance.^4 5^ Similarly, guidelines for neck pain recommend education that focuses on reassurance of recovery, encouragement to remain active, pain management strategies and prognosis.^6^ However, emerging evidence suggests that guideline-based education does not always align with patients’ needs. A mixed-methods study on acute low back pain^28^ found that patients often have a range of concerns (eg, the causes of and the future consequences of low back pain) that are not addressed by guideline-recommended education. Therefore, aligning education with patients’ expectations and delivering information that is individually relevant to their condition may help enhance reassurance and improve self-efficacy for rotator cuff-related shoulder pain.

### Unanswered questions and future research

While our findings support the potential benefits of *best practice education* (with or without *pain science messages*) in reducing interest in unnecessary treatments and promoting self-management, its long-term effectiveness remains unknown. In particular, it is unclear whether adding *pain science messages* meaningfully enhances the effects of *best practice education* alone, as both the quantitative and qualitative differences observed in our studies were small. Future trials embedded in clinical practice are recommended as an important next step for evaluating the effectiveness of different advice and education for shoulder pain.

It also remains unclear whether *best practice education* (with or without *pain science messages*) improves patient-reported clinical outcomes such as pain, function and quality of life. These approaches have shown benefit for other musculoskeletal conditions, including back pain and neck pain,^29 30^ but comparable evidence in shoulder pain is lacking.^31^ To date, only one trial has investigated this—the trial comparing *best practice education* (with or without *pain science messages*) to *structure-focused education* (from our quantitative experiment). Therefore, there is a need to evaluate the effects of other educational strategies.

## Conclusion

Participants with rotator cuff-related shoulder pain expressed generally similar emotional responses across groups, with small differences in treatment preferences favouring self-management in the *best practice education* groups compared with those who received *structure-focused education*. Those in the *best practice education* also less frequently reported needing potentially unnecessary treatments (eg, imaging, injections and surgery).

## Supplementary material

10.1136/bmjopen-2025-107032 online supplemental file 1

10.1136/bmjopen-2025-107032 online supplemental file 2

10.1136/bmjopen-2025-107032 online supplemental file 3

## Data Availability

Data are available upon reasonable request.
