# Supplementary material for: Perceptions of best practice, pain science and structure-focused education for rotator cuff-related shoulder pain: a content analysis of qualitative data from a randomised experiment
Source: BMJ Open. 2026 Feb 6;16(2):e107032. doi: 10.1136/bmjopen-2025-107032 (PMC12887478; doi:10.1136/bmjopen-2025-107032)
Supplement: online supplemental file 1 [file bmjopen-16-2-s001.docx]

**SUPPLEMENTARY FILE 1. Welcome to the Study, Consent, Screening, And Survey**

**Welcome to the Advice about Shoulder Pain study!**

Thank you for your interest in taking part in our study on shoulder pain. Researchers at the University of Sydney are doing this study to understand what people think about different patient education for shoulder pain. The survey will take approximately 15-20 minutes. Our researchers take your privacy very seriously and all responses will be anonymous. You can also exit from the survey at any time and your responses will be deleted if you do not complete the survey.

Please click on the link below to see and get a Participant Information Sheet. This will

explain more about the study. Please read it carefully before making up your mind about taking part. If you have any questions, please get in touch with one of the research team using the phone numbers or emails listed in the information sheet.

You must be 18 years or older to take part in this study.

[Participant Information Statement](https://unisyd-my.sharepoint.com/personal/zixin_zhang_sydney_edu_au/Documents/Content%20Analysis%20Education%20Shoulder/Submission/BMJ%20Open/APPENDIX-1.-Participant-Information-Statement_v1_300123.docx?web=1)

Once you have read the Participant Information Sheet, please click the next button to get the consent form and to start the survey.

Thank you for supporting this important research.

**PARTICIPANT CONSENT FORM**

**Advice about shoulder pain study**

**In giving my consent I acknowledge that:**

| **✓** | I have read the Participant Information Statement and have been given the opportunity to discuss the study and my involvement in it with the researcher/s. |
| --- | --- |
| **✓** | The procedures required and time involved (including any inconvenience, risk, discomfort or side effect, and their implications) have been explained to me, and my questions about the project have been answered to my satisfaction. |
| **✓** | I understand that participation is voluntary. I am under no obligation to consent. |
| **✓** | I understand that I can withdraw from the study at any time, without providing a reason and without suffering any penalty. This will not affect my relationship with the researcher/s or university. |
| **✓** | I understand that my involvement is strictly confidential and no information about me will be used in any way that reveals my identity. |
| **✓** | I understand that data from this study may be used again for future research purposes, but that all data is strictly confidential and no information about me will be used in any way that reveals my identity. |

- **Yes, I would be happy to go on and complete the survey**
- **No, I would prefer not to complete the survey**

**SCREENING QUESTIONS**

1. **Do you currently have any of the following: [people who do not select ‘pain in the shoulders’ or select all of the options will be excluded]**
   1. Headache
   2. Pain in your neck
   3. Pain in your shoulder(s)
   4. Pain in your lower back
   5. Pain in your hip(s)
   6. Pain in your stomach
   7. Pain in your knee(s)
   8. None of the above
2. **On average, how much shoulder pain have you had over the past week? [people who select ‘0’ will be excluded]**

0 1 2 3 4 5 6 7 8 9 10

No pain at all Worst pain imaginable

1. **Is your shoulder pain mainly experienced within the shaded area as shown in the photos below (on either your left or right shoulder)?** [people who select ‘No’ will be excluded]
   1. Yes
   2. No
2. **Have you been told by a health professional (e.g. doctor, physiotherapist) that you have any of the following shoulder issues? Please select the ones you’ve had or currently have [people who select any of the issues below will be excluded]**
   1. Frozen shoulder (‘adhesive capsulitis’)
   2. Osteoarthritis in the shoulder
   3. Shoulder instability (i.e. loose ligaments in the shoulder)
   4. A shoulder fracture
   5. A shoulder dislocation
   6. Cancer in the shoulder
   7. Infection in the shoulder
   8. I have not had any of these conditions

**SURVEY**

**First some quick questions about you...**

**Are you:**

- Female
- Male
- Non-binary
- Prefer not to say

**Please indicate your age:** [free text response]

**In which country were you born?** [free text response]

**What option best describes your highest level of education?**

- Primary school or less
- High school (not completed)
- High school (completed)
- TAFE/Trade
- University- undergraduate degree/s (completed)
- University- postgraduate degree/s e.g. Masters, PhD (completed)
- Other (please specify) ____________________________

**What is your employment status?**

- Employed part-time
- Employed full-time
- Casual work
- Retired
- Unemployed
- Student
- Sick/disability leave
- Other (please specify) ____________________________

**Do you have private health insurance?**

- Yes
- No

**Now some questions about your shoulder pain...**

**How long have you had your current shoulder pain?**

- Less than 1 week
- 1 to 3 months
- 4 months to 12 months
- Longer than 12 months
- Not applicable. I do not have shoulder pain [Exclude from survey]

**Have you previously had a scan (e.g. X-ray, ultrasound, MRI) for shoulder pain?**

- Yes
- No

**Have you previously seen a physiotherapist for your shoulder pain?**

- Yes
- No

**Have you previously had an injection into the shoulder for your pain?**

- Yes
- No

**Have you previously had shoulder surgery?**

- Yes
- No

**Have you previously taken sick leave (time off work) due to shoulder pain?**

- Yes
- No

**Have you ever received advice about shoulder pain from a health professional?**

- Yes
- No (skip next two questions)

**Based on this advice, what is your understanding about what causes shoulder pain?** [free text response]

**Based on this advice, what is your understanding about the best treatment for shoulder pain?** [free text response]

**Shoulder Pain and Disability Index (SPADI)**

Please read carefully.

Instructions: Please circle the number that best describes the question being asked.

*Pain scale (don’t respond to this scale):*

*0 1 2 3 4 5 6 7 8 9 10*

*No pain at all Worst pain imaginable*

**How severe is your pain?**

1. At its worst?

0 1 2 3 4 5 6 7 8 9 10

2. When lying on the involved side?

0 1 2 3 4 5 6 7 8 9 10

3. Reaching for something on a high shelf?

0 1 2 3 4 5 6 7 8 9 10

4. Touching the back of your neck?

0 1 2 3 4 5 6 7 8 9 10

5. Pushing with the involved arm?

0 1 2 3 4 5 6 7 8 9 10

*Disability scale (don’t respond to this scale):*

*0 1 2 3 4 5 6 7 8 9 10*

*No difficulty So difficult it requires help*

**How much difficulty do you have?**

1. Washing your hair?

0 1 2 3 4 5 6 7 8 9 10

2. Washing your back?

0 1 2 3 4 5 6 7 8 9 10

3. Putting on an undershirt or pullover/sweater?

0 1 2 3 4 5 6 7 8 9 10

4. Putting on a shirt that buttons down the front?

0 1 2 3 4 5 6 7 8 9 10

5. Putting on your pants?

0 1 2 3 4 5 6 7 8 9 10

6. Placing an object on a high shelf?

0 1 2 3 4 5 6 7 8 9 10

7. Carrying a heavy object of 10 pounds (~4.5kgs)?

0 1 2 3 4 5 6 7 8 9 10

8. Removing something from your back pocket?

0 1 2 3 4 5 6 7 8 9 10

**How tense or anxious have you felt in the past week?**

*0 1 2 3 4 5 6 7 8 9 10*

*Not at all Extremely*

**How much have you been bothered by feeling depressed in the past week?**

*0 1 2 3 4 5 6 7 8 9 10*

*Not at all Extremely*

**How much ‘fear’ do you have that your shoulder pain would be increased by physical activity?**

*0 1 2 3 4 5 6 7 8 9 10*

*No fear A great deal of fear*

**[Participants will then be randomised to one of three interventions, more details can be found in elsewhere (**[**https://journals.lww.com/pain/fulltext/2024/04000/education_can_reassure_people_with_rotator.20.aspx**](https://journals.lww.com/pain/fulltext/2024/04000/education_can_reassure_people_with_rotator.20.aspx)**). After they view the video and read the script, they will be asked the following questions]**

**Think carefully about the advice you received and answer the following questions…**

**Reassurance**

**Based on the advice you received, how reassured do you feel that there is no serious condition causing your shoulder pain?**

0 1 2 3 4 5 6 7 8 9 10

Not reassured at all Completely reassured

**Based on the advice that you received, how reassured do you feel that continuing with your daily activities is safe?**

1. 1 2 3 4 5 6 7 8 9 10

Not reassured at all Completely reassured

**What message(s) from the advice reassured you? If the advice did not reassure you at all, write N/A in the text box below.** [free-text response]

**Similarity to previous advice**

**Is the advice you just received similar to the advice previously given to you by a health professional(s)?**

- Yes
- No
- Unsure
- I have no received advice from a health professional before

**Treatment intentions**

**Based on the advice that you received, do you intend to stay active for your shoulder pain?**

0 1 2 3 4 5 6 7 8 9 10

Definitely do Definitely do not

**Based on the advice that you received, do you intend to see a health professional for your shoulder pain?**

0 1 2 3 4 5 6 7 8 9 10

Definitely do Definitely do not

**Based on the advice that you received, do you intend to see a surgeon for your shoulder pain?**

0 1 2 3 4 5 6 7 8 9 10

Definitely do Definitely do not

**Based on the advice that you received, do you intend to get a shoulder scan (e.g. X-Ray, MRI)?**

0 1 2 3 4 5 6 7 8 9 10

Definitely do Definitely do not

**Credibility of the advice**

1. **At this point, how logical does the advice offered to you seem?**

0 1 2 3 4 5 6 7 8 9 10

Not at all logical Somewhat logical Very logical

1. **At this point, how successfully do you think this advice will be in reducing your shoulder pain?**

0 1 2 3 4 5 6 7 8 9 10

Not at all useful Somewhat useful Very useful

1. **How confident would you be in recommending this treatment to a friend who experiences similar problems?**

0 1 2 3 4 5 6 7 8 9 10

Not at all confident Somewhat confident Very confident

1. **After receiving this advice, how much improvement in your shoulder pain do you think will occur?**

0% 10% 20% 30% 40% 50% 60% 70% 80% 90% 100%

**Relevance of the advice**

1. To what extent did the information that you received address your concerns?

0 1 2 3 4 5 6 7 8 9 10

Did not address my concerns at all Addressed my concerns completely

**Feelings and treatment needs evoked by the advice**

**What did you like about the advice you received?** [free-text response]

**What did you dislike about the advice you received?** [free-text response]

**If your health professional gave you this advice, how would it make you feel?** [free-text response]

**If your health professional gave you this advice, what treatments (if any) do you think you would need?** [free-text response]
