# Supplementary material for: Perceptions of best practice, pain science and structure-focused education for rotator cuff-related shoulder pain: a content analysis of qualitative data from a randomised experiment
Source: BMJ Open. 2026 Feb 6;16(2):e107032. doi: 10.1136/bmjopen-2025-107032 (PMC12887478; doi:10.1136/bmjopen-2025-107032)
Supplement: online supplemental file 2 [file bmjopen-16-2-s002.docx]

**Supplementary File 2. Coding Frameworks**

**Question 1. *If your health professional gave you this information, how would it make you feel?***

| **Code** | **Explanation** | **Examples** |
| --- | --- | --- |
| Activity restriction | Any reference to being unable to do typical daily activities | Caution, light work, rest, sleep loss, time off work, careful |
| Avoid treatment/investigation | Any reference to avoiding treatment or investigation | Avoid surgery |
| Aging | Any reference to the condition being due to aging | Old, getting old/older, ancient |
| Contrasting experience | Any reference to previously experiencing a different outcome than outlined in the advice | Advice gives a worse outlook than a previous experience I had, it hasn't helped the pain |
| Empowered | Any reference to having control, power or knowledge over how to manage their shoulder pain | Confident, knowledge, informed, educated, in control of their life, make decisions |
| Feeling dismissed | Any reference to feeling dismissed | Didn’t listen to me, not interested in my opinion, minimising my issue |
| Good prognosis | Any reference to the condition recovering either quickly or without treatment, or feeling optimistic about their future | Temporary, no treatment needed, heal over time, optimistic, positive about recovery, the problem can be addressed |
| Have received similar advice before | Any reference to having received similar advice previously | Already know, heard this before |
| Irrelevant response | The response did not address the question | I don’t have any feelings |
| Mechanism of injury | Any reference to why the pain started | Injury, overuse issue, caused by lifting, sports injury |
| Minor issue | Any reference to the condition being ‘non-serious’ | Not serious, everyday issue, common, annoyance, uncomfortable, inconvenient |
| Need more information or options | Any reference to needing more information or options | I need more information, the next step after hearing the advice |
| Negative about the advice | Any negative comment about the content of the advice | Too brief, doctor is an idiot, sceptical, doctor doesn’t know what he’s talking about, wasting time and money, common knowledge, generalised |
| Negative about the tone or presentation of the advice | Any negative comments about the tone or presentation of the advice | Cold and uncaring, heartless |
| No impact on thoughts and/or feelings | Any reference to the advice not impacting their thoughts or feelings | Nothing, the same |
| Attention to pain | Any reference to their shoulder pain | Hurt, intermittent, discomfort, recurrent |
| Poor prognosis | Any reference to the condition taking a long time to recover | Persistent pain, long recovery, long-term issue |
| Positive about the advice | Any positive comment about the content of the advice | Good advice, makes sense, useful, makes me feel supported, helped a lot, helpful, feel good, very positive, encouraged |
| Psychological distress | Any reference to feelings of fear, anxiety, worry or stress | Fear, anxious, worry, stress, scared, depressed, nervous, etc. |
| Reassurance | Any reference to feelings of reassurance, relief or happiness | Relieved, comforted, happy, calm |
| Second opinion | Any reference to needing a second opinion | Check with my doctor |
| Serious issue | Any reference to the condition being ‘serious’ | Deteriorating, serious, bad, very ill |
| Tissue damage or dysfunction | Any reference to tissue damage or dysfunction | Tendon tear, arm out of place, sprained ligaments, pulled muscle, stiffness, weakness |
| Treatment/investigation | Any reference to the need for treatment or investigation | Rest, pain medication, heat, surgery, physiotherapy, requires imaging, urgent treatment |
| Trust in expertise | Any reference to trusting the advice because it was delivered by a healthcare professional | I trust the advice because its coming from a surgeon, reliable, believable, very professional, they knew what they are talking about |
| Uncertainty | Any reference to being unsure what the advice means | Complicated, confused, uncertainty, feel skeptical |
| Unhappy/frustrated | Any reference to being unhappy or frustrated | Sad, anger, annoyed, feel bad, upset, helpless, useless |
| Willing to follow the advice | Any reference to being willing to follow the advice | Proceed to follow it, give it a try, think about it, accept it, being aware |
| Validated or cared for | Any reference to feeling validated or cared for | Understood, caring, empathetic, heard |

***Question 2. If your health professional gave you this information, what treatments (if any) do you think you would need?***

| **Code** | **Examples (if needed)** |
| --- | --- |
| Activity modification | Avoid lifting, avoid aggravating activities, avoid strenuous activities |
| Acupuncture |  |
| Chiropractor |  |
| Psychological therapies | Cognitive behavioural therapy, see a psychiatrist |
| Cold |  |
| Compression |  |
| Diet |  |
| Doctor |  |
| Education/advice |  |
| Elevation |  |
| Emergency department/hospital |  |
| Ergonomics/posture | Adjust computer screen height |
| Exercise |  |
| Follow up appointments |  |
| Follow the advice provided |  |
| Good mattress or pillows |  |
| Heat |  |
| Immobilisation | Sling |
| Injection | Cortisone injection |
| Investigations | X-ray, ultrasound, MRI |
| Light exercise | Gentle exercise, exercise but be careful, stretching |
| Manipulation |  |
| Massage |  |
| Medication | Panadol, anti-inflammatories, muscle relaxants, supplements |
| Irrelevant response |  |
| Natural or unknown therapies | Stone therapy, finger therapy, natural remedies, tea, spa baths |
| Normal movements | Keep arm moving, normal activity, stay active |
| Osteopathy |  |
| Physiotherapy |  |
| Plan | Treatment plan, recovery plan |
| Prayer/hope/meditation |  |
| Rest | Taking it easy, relaxation, reduce overall activity |
| Second opinion |  |
| Social support | Family, Friends |
| Specialist |  |
| Stay healthy | Good sleep, avoid smoking |
| Surgery |  |
| Taping/bracing | Brace, strapping, sling |
| Time off work |  |
| Topical treatments | Ointment, rub, Voltaren gel, oils |
| Treatment unspecified | Refers to the need for treatment but does not specify what’s needed |
| Unsure |  |
| Wait and see | Time, monitor, no treatment |
| Therapy |  |
