# Supplementary material for: Perceptions of best practice, pain science and structure-focused education for rotator cuff-related shoulder pain: a content analysis of qualitative data from a randomised experiment
Source: BMJ Open. 2026 Feb 6;16(2):e107032. doi: 10.1136/bmjopen-2025-107032 (PMC12887478; doi:10.1136/bmjopen-2025-107032)
Supplement: online supplemental file 3 [file bmjopen-16-2-s003.docx]

**Supplementary File 3. Examples of participants’ open-ended responses regarding ‘words or feelings’ (question 1) by Education**

| Group 1 | Group 2 | Group 3 |
| --- | --- | --- |
| **Activity restriction** | | |
| [No examples] | “I do feel good as long as I do not over do things”  *[Female, early 50s]*  “I feel like I'll take a break”  *[Male, mid 30s]* | “I'd be making changes to my lifestyle”  *[Female, early 60s]*  “Feel like I need some time off”  *[Male, early 30s]* |
| **Aging (only female responses)** | | |
| “There was probably no cure - just ageing”  *[Female, early 80s]* | “It does help as I know I am getting older”  *[Female, early 50s]* | “The cause is my age”  *[Female, mid 60s]* |
| **Attention to pain** |  |  |
| “I had been experiencing the pain for a long time”  *[Female, mid 30s]*  “Not very optimistic about the pain”  *[Male, early 60s]* | “My pain persisted”  *[Female, late 20s]*  “The pain comes and goes”  *[Male, early 40s]* | “I am in pain in different places”  *[Female, late 40s]*  “I have spent 8 unnecessary years in pain”  *[Male, early 70s]* |
| **Avoid treatment/investigation** | | |
| “Relieved I don’t require surgery”  *[Female, late 60s]*  “Surgery is not required”  *[Male, early 60s]* | “I had more options and do not have to resort to extreme measures of treatment like surgery”  *[Female, mid 30s]*  “My pain wasn’t in need of surgery”  *[Female, mid 40s]* | “Surgery will be avoided”  *[Female, mid 60s]*  “I don't need to see health professionals”  *[Male, late 50s]* |
| **Contrasting experience** | | |
| “I've heard it all already and it hasn't helped the pain”  *[Female, late 70s]*  [only one example in this group] | “I tried it all years ago and found it to be useless”  *[Female, mid 60s]*  “I did get similar advice, and it worsened the pain”  *[Male, early 50s]* | [No examples] |
| **Empowered** | | |
| “It would make me feel empowered”  *[Female, mid 50s]*  “More confident about using my shoulder”  *[Male, mid 50s]* | “I know how to take care of my body”  *[Female, late 30s]*  “Confident I can handle my shoulder pain when it is more severe”  *[Male, early 30s]* | “Empowered with knowledge and advice”  *[Female, early 70s]*  “It would make me feel more knowledgeable if my other shoulder experiences some kind of pain”  *[Male, early 30s]* |
| **Feels dismissed** | | |
| “Not fully cared for”  *[Female, early 50s]*  “Placated in a patronizing way”  *[Male, late 40s]* | “A little fobbed off”  *[Female, early 40s]*  “Insulated”  *[Male, mid 60s]* | “Slightly dismissed”  *[Female, early 50s]*  “I would not feel heard”  *[Female, late 40s]* |
| **Good prognosis** | | |
| “I would feel like more than likely my issue would pass”  *[Female, late 20s]*  “I'll get better soon”  *[Male, late 40s]* | “I feel like I have a cure”  *[Female, late 20s]*  “I think I'll get better soon”  *[Male, early 40s]* | “Positive for the future”  *[Female, early 40s]*  “I feel more hopeful and confident that the pain will get relief”  *[Male, early 30s]* |
| **Have received similar advice before** | | |
| “My doctor did give me advice like this”  *[Female, mid 30s]*  “He did give me similar advice”  *[Male, late 50s]* | “I did receive this advice, and it explained my pain”  *[Female, early 70s]*  “I did get similar advice”  *[Male, early 50s]* | “My health professionals already gave me similar advice”  *[Female, mid 60s]*  “It's something I've already heard”  *[Male, early 50s]* |
| **Irrelevant response** | | |
| “Don’t know”  *[Female, early 40s]*  “Not sure”  *[Male, early 40s]* | “Unsure”  *[Female, mid 60s]*  “None”  *[Male, early 30s]* | “Not sure”  *[Female, early 60s]*  “No comment”  *[Male, early 70s]* |
| **Mechanism of injury** | | |
| “I would still want to know the cause of the pain and the extent of the injury”  *[Female, late 50s]*  “A little disappointed that a cause couldn't be pinpointed”  *[Male, mid 50s]* | “Chronic poor posture and work stress”  *[Male, mid 30s]*  [only one example in this group] | “My condition was caused by a repetitive strain injury”  *[Female, mid 60s]*  [only one example in this group] |
| **Minor issue** | | |
| “Relieved it may not be serious”  *[Female, early 50s]*  “I don't think it's a big deal”  *[Male, early 30s]* | “My shoulder pain is not as serious as I may think”  *[Female, late 20s]*  “There's nothing serious with my shoulder”  *[Male, early 40s]* | “It reassures me that it isn’t more serious”  *[Female, early 50s]*  “Relieved to know it was not serious”  *[Male, late 70s]* |
| **Need more information or options** | | |
| “I would want to find out more about my shoulder”  *[Female, late 30s]*  “I would expect greater detail”  *[Male, early 50s]* | “Like I need to think and get more info”  *[Female, early 40s]*  “I would definitely have more questions about why they feel this way”  *[Male, early 30s]* | “I would need more information”  *[Female, late 50s]*  “I may wish to seek more information and professional advice to make an informed decision”  *[Male, late 40s]* |
| **Negative about the advice** | | |
| “Bored and uninterested”  *[Female, early 30s]*  “It is generally useless”  *[Male, mid 50s]* | “I feel like some of it was common knowledge”  *[Female, late 20s]*  “He did not know what is wrong”  *[Male, mid 70s]* | “Too generalized for my specific needs”  *[Female, early 30s]*  “I wasted money on a doctor visit”  *[Male, late 40s]* |
| **Negative about the tone or presentation of the advice** | | |
| “They didn’t actually care”  *[Female, late 20s]* | [No examples] | [No examples] |
| **No impact on thoughts and/or feelings** | | |
| “Neither satisfies or dissatisfied”  *[Female, early 20s]*  “The same as before”  *[Male, early 50s]* | “No big change in my behaviour”  *[Female, late 60s]*  “Same as now”  *[Male, mid 60s]* | “The same”  *[Female, late 60s]*  “Same”  *[Male, mid 60s]* |
| **Poor prognosis** | | |
| [No examples] | “Fibro never heals”  *[Female, early 30s]* | “It could have been worse”  *[Male, mid 60s]* |
| **Positive about the advice** | | |
| “Made me feel supported”  *[Female, late 30s]*  “I would be satisfied with the level of detail”  *[Male, late 30s]* | “Mostly Good”  *[Female, early 40s]*  “It would make me feel great”  *[Male, late 40s]* | “I think it's very useful”  *[Female, mid 30s]*  “That what they were saying made sense”  *[Male, mid 50s]* |
| **Psychological distress** | | |
| “Very concerned”  *[Female, early 70s]*  “Well concerning”  *[Male, early 70s]* | “More depression and misery”  *[Female, mid 60s]*  “I would be very worried”  *[Male, late 40s]* | “I would feel serious and worried”  *[Female, mid 30s]*  “Maybe a little concerned”  *[Male, late 60s]* |
| **Reassured** | | |
| “Calm and reassured”  *[Female, mid 40s]*  “It would made me feel reassured”  *[Male, early 40s]* | “Definitely assured”  *[Female, late 30s]*  “Put my mind at ease”  *[Male, early 30s]* | “I am very relieved”  *[Female, late 30s]*  “I feel more comfortable”  *[Male, early 40s]* |
| **Second opinion** | | |
| “I always seek advice from multiple healthcare professions and professionals”  *[Female, late 20s]*  “I would seek a second opinion”  *[Male, early 40s]* | “I would be seeking confirmation from other sources”  *[Female, mid 50s]*  “I would go to another doctor”  *[Male, early 50s]* | [No examples] |
| **Tissue damage or dysfunction** | | |
| “My issue is connected with nerve damage”  *[Male, early 70s]* | [No examples] | [No examples] |
| **Treatment/investigation** | | |
| “I would want to at least have an Xray to rule out anything going on”  *[Female, early 50s]*  “I may want to get an X-ray or MRI”  *[Male, late 50s]* | “I would still ask for an x-ray”  *[Female, late 30s]*  “Want a pill or therapy”  *[Male, late 60s]* | “I feel that it may require some x-rays”  *[Female, mid 50s]*  “It was nothing really serious as long as I took the medication provided”  *[Male, early 60s]* |
| **Trust in expertise** | | |
| “I feel it is reliable”  *[Female, mid 30s]*  “More believable”  *[Male, early 50s]* | “I would trust it”  *[Female, late 20s]*  “Having professional help can make me feel more at ease”  *[Male, late 30s]* | “I would be reasonable to hear the advice from the expert”  *[Female, late 20s]*  “I was with a health professional that knew what they were talking about”  *[Male, mid 40s]* |
| **Uncertainty** | | |
| “Not real sure of his advice”  *[Female, mid 60s]*  “I may feel confused due to inconsistencies and uncertainties in medical information. I may not be sure how to make decisions”  *[Male, early 30s]* | “It would still leave me with a few questions”  *[Female, early 40s]*  “Somewhat sceptical”  *[Male, mid 60s]* | “Not sure what can be done to address the problem”  *[Female, mid 60s]*  “I would likely feel a little confused about what treatment would be best for me”  *[Male, late 30s]* |
| **Unhappy/frustrated** | | |
| “Pretty annoyed to be getting unhelpful advice”  *[Female, late adolescence]*  “A little unhappy there's no quicker fix”  *[Male, early 30s]* | “It would make me annoyed”  *[Female, mid 30s]*  “Angry”  *[Male, early 50s]* | “Not happy”  *[Female, late 50s]*  “In a bad mood”  *[Male, early 50s]* |
| **Willing to follow the advice** | | |
| “I will follow the advice”  *[Female, late 20s]*  “I would follow his advice to see if it helps”  *[Male, late 50s]* | “I would listen and do as told”  *[Female, late 30s]*  “That's very good to try”  *[Male, early 30s]* | “I would try her recommendations first”  *[Female, mid 60s]*  “I will trust their expertise and follow their advice”  *[Male, mid 30s]* |
| **Validated or cared for** | | |
| “Heard and listened to”  *[Female, late 20s]*  “Valued and appreciated”  *[Male, late 20s]* | “They'd listened to my concerns”  *[Female, mid 40s]*  “Well looked after”  *[Male, late 60s]* | “At least heard instead of ignored”  *[Female, early 50s]*  “Understood and helpful”  *[Male, late 60s]* |
