# Supplementary material for: Perceptions of best practice, pain science and structure-focused education for rotator cuff-related shoulder pain: a content analysis of qualitative data from a randomised experiment
Source: BMJ Open. 2026 Feb 6;16(2):e107032. doi: 10.1136/bmjopen-2025-107032 (PMC12887478; doi:10.1136/bmjopen-2025-107032)
Supplement: Supplementary file 4 [file bmjopen-2025-107032.reviewer_comments.pdf]

PEER REVIEW HISTORY

BMJ Open publishes all reviews undertaken for accepted manuscripts. Reviewers are asked to complete a checklist review form and are provided with free text boxes to elaborate on their assessment. These free text comments are reproduced below.

ARTICLE DETAILS

Title (Provisional)

Perceptions of best practice, pain science, and structure-focused education for rotator cuff-related shoulder pain: a content analysis of qualitative data from a randomised experiment

Authors

Zhang, Zixin; Ferreira, Giovanni E; Muller, Ryan; Sousa, Fernando; Malliaras, Peter; West, Courtney A; O'Keeffe, Mary; Maher, Chris; Zadro, Joshua

---

VERSION 1 - REVIEW

**Reviewer**	**1**
**Name**	**Vishal, Kavitha**
**Affiliation**	**Manipal College of Health Professions, Department of Physiotherapy**
**Date**	**24-Aug-2025**
**COI**	**None**

This remains a strong, innovative study that addresses a gap in patient education for rotator cuff-related shoulder pain. The qualitative content analysis of your randomized experiment provides valuable insights into emotional responses and treatment perceptions, with implications for reducing low-value care (unnecessary imaging/surgery). However, there are areas where the manuscript could be improved to enhance clarity, rigor and generalizability. I recommend revisions to address these concerns, as detailed below

Major concerns:

1. The manuscript describes a screening process to RCRSP diagnosis, but it acknowledges the limitation of online data collection. The feasibility of confirming RCRSP online is limited but possible with carefully designed screening questions that mimic clinical criteria. The inclusion of pain location and exclusion of other diagnoses is a strength, as it reduces the likelihood of including non-RCRSP conditions. However, the reliance on self-reported “no diagnosis” for exclusion criteria is a weakness, as participants may not recall or know their diagnoses accurately. Participants may misinterpret pain location or recall diagnoses

incorrectly. Moreover, the broad inclusion criteria (pain ≥1/10, any duration) include mild or acute cases unlikely to reflect clinical RCRSP populations seeking care.

Expand the limitation to explicitly state the risk of misclassification due to self-reported diagnosis. Discuss how reliance on participant recall (e.g., “no diagnosis of adhesive capsulitis”) may miss undiagnosed cases.

Clarify why other symptom characteristics were omitted in the methods.

2. The manuscript does not address how the coders’ backgrounds such as the expertise in physiotherapy, the MPH students’ public health perspective, or ZZ’s focus on sports medicine may have influenced code development or application. Additionally, describe the coders’ prior experience with handling qualitative data

3. Was there a metric decided for coding agreement?

4. Content analysis appears appropriate, but the responses appear short limiting depth. Did you add any sub-themes or have any contextual nuances, given that your sample was large enough to capture that?

5. The manuscript doesn't detail what that prior education entailed (e.g., content, source, physiotherapy, GP, online resources biomedical/structure-focused vs. biopsychosocial/best practice). This lack of clarity is a key gap, as the type of prior education matters more than its mere presence. Repeated exposure to similar ideas could shrink group differences as participants are most likely to provide familiar responses derived from the previous education especially when responses were taken immediately.

Minor comments:

Abstract: Page 5 of abstract in the conclusion, the sentence that the participants who received best practice education preferred self -management strategies could be clarified by a percentage or a qualitative descriptor

The phrase “generally similar emotional responses and treatment needs” could be more specific. For example, revise to: “Similar emotional responses across groups, with small differences in treatment preferences favoring self-management in best practice groups.

Page 9, methods section- Clarify whether the length of the video varied between the groups.

Page 19- The term “overmedicalization” in the summary appears without a definition or citation. This term may not be familiar to the readers.

**Reviewer**	**2**
**Name**	**Willems, WJ**
**Affiliation**	**De Lairesse Kliniek, Amsterdam, orthopedics**
**Date**	**10-Nov-2025**

COI **None**

This study is quite interesting, to evaluate the effect of patient information on patient's perception.

The both questions are adequate. Given the fact, that most guidelines are rather one sided regarding patient information.

The number of participants is impressive.

There are some questions.

-Patients were selected from market research panels. They were asked if a diagnosis of the exclusion list was made in the previous period. This might include a bias: patients with e.g. shoulder arthritis or capsulitis could be involved in the study, not (yet) being diagnosed by a health professional.

-Looking to the numbers in table 2 and 3, the differences between the 3 types of education in the 30 items (table 2) and 40 items (table 4) are not impressive.

Which statistical program was used for this study ?

It is difficult to agree with the conclusions, considering the small differences between the 3 groups.

It is recommended, that the authors explain, why these small differences are significant.

One of the weaknesses of this study is the online presentation of the question.

Did the authors consider a(smaller) randomised trial between this online questioning versus a face to face questioning?

VERSION 1 - AUTHOR RESPONSE

Detailed Comments from Reviewer #1:

Reviewer: 1

Dr. Kavitha Vishal, Manipal College of Health Professions

Comments to the Author:

This remains a strong, innovative study that addresses a gap in patient education for rotator cuff-related shoulder pain. The qualitative content analysis of your randomized experiment provides valuable insights into emotional responses and treatment perceptions, with implications for reducing low-value care (unnecessary

imaging/surgery). However, there are areas where the manuscript could be improved to enhance clarity, rigor and generalizability. I recommend revisions to address these concerns, as detailed below

AUTHOR'S RESPONSE:

Thank you for the kind words and taking the time to provide this thorough review, we have revised our manuscript based on your comments.

Major concerns:

1. The manuscript describes a screening process to RCRSP diagnosis, but it acknowledges the limitation of online data collection. The feasibility of confirming RCRSP online is limited but possible with carefully designed screening questions that mimic clinical criteria. The inclusion of pain location and exclusion of other diagnoses is a strength, as it reduces the likelihood of including non-RCRSP conditions. However, the reliance on self-reported "no diagnosis" for exclusion criteria is a weakness, as participants may not recall or know their diagnoses accurately. Participants may misinterpret pain location or recall diagnoses incorrectly. Moreover, the broad inclusion criteria (pain ≥1/10, any duration) include mild or acute cases unlikely to reflect clinical RCRSP populations seeking care.

Expand the limitation to explicitly state the risk of misclassification due to self-reported diagnosis. Discuss how reliance on participant recall (e.g., "no diagnosis of adhesive capsulitis") may miss undiagnosed cases.

AUTHOR'S RESPONSE:

We agree with the reviewer that diagnosing RCRSP online, while attempting to exclude specific and serious shoulder pathologies, is challenging. We have now added the following to the limitations section:

Limitations

Another limitation is how we identified participants with rotator cuff-related shoulder pain. Our screening questions included participants if they self-identified as currently having shoulder pain with pain over the past week ≥1 on a scale of 0-10, had shoulder

pain around the anterolateral part of the shoulder and upper arm, and reported that they had not been diagnosed by a health professional with other specific or serious shoulder pathologies. These screening questions may be prone to recall bias if participants did not recall the specific or serious diagnosis they previously received from a health professional and may have captured some people with a specific diagnosis if they had not been formally diagnosed by a health professional. These inclusion criteria also likely captured some mild cases that may not reflect a clinical or care-seeking population. However, we consider this a minimal limitation because the mean pain scores in our sample are largely similar to other trials of people with shoulder pain and are therefore likely to be representative of the broader literature [19, 25-28].

Clarify why other symptom characteristics were omitted in the methods.

AUTHOR'S RESPONSE:

2. Thank you for this suggestion. We have now clarified why other symptom characteristics were omitted in the methods:

Data collection

… We did not capture further information about symptom characteristics to reduce the burden of these screening questions on participants and since our screening criteria approximate a diagnosis of rotator cuff-related shoulder pain [12].

2. The manuscript does not address how the coders' backgrounds such as the expertise in physiotherapy, the MPH students' public health perspective, or ZZ's focus on sports medicine may have influenced code development or application. Additionally, describe the coders' prior experience with handling qualitative data

AUTHOR'S RESPONSE:

Thank you for noting this. We have now clarified these issues in the methods:

Data analysis

An additional PhD student with a background in sports medicine (ZZ) independently reviewed the responses to triple code the responses and to identify discrepancies in coding against the framework. ZZ had some experience in coding content analyses prior to this study but was supervised by JZ who has extensive experience in content analysis [15-17]. All responses were coded independently, and all disagreements resolved through further consultation with JZ. All coders were blinded to group allocation.

The coders' expertise mostly relates to conservative management of rotator cuff-related shoulder pain, which may have influenced the lens by which the data were interpreted. However, the use of double coding and discussion between coders strengthened the trustworthiness of the analysis, and the coding framework adapted from previous studies [15, 17, 18] likely reduced any subjective interpretations related to the coders background and expertise.

3. Was there a metric decided for coding agreement?

AUTHOR'S RESPONSE:

Thank you for noting this. We did not use a metric for coding agreement as all responses were double or triple coded and any disagreements were resolved through discussion. Calculating a metric for agreement (e.g., kappa) is only necessary when responses are coded by one researcher. See previous studies employing our methodology:
https://bmjopen.bmj.com/content/14/7/e079070;
https://link.springer.com/article/10.1007/s00586-022-07365-x#Sec2

4. Content analysis appears appropriate, but the responses appear short limiting depth. Did you add any sub-themes or have any contextual nuances, given that your sample was large enough to capture that?

AUTHOR'S RESPONSE:

Thank you for pointing this out. We agree responses were short and have mentioned this as a limitation:

"Most responses were short (see Supplementary File 3), which may have limited the depth of the data we found."

We did not create any sub-themes given most responses were quite short and there was a relatively small number of codes overall. This is consistent with previous content analyses in the field which have just summarised codes and not structured the results as themes/sub-themes which would be more appropriate for more in-depth data:

https://bmjopen.bmj.com/content/14/7/e079070;
https://link.springer.com/article/10.1007/s00586-022-07365-x#Sec2

5. The manuscript doesn't detail what that prior education entailed (e.g., content, source, physiotherapy, GP, online resources biomedical/structure-focused vs. biopsychosocial/best practice). This lack of clarity is a key gap, as the type of prior education matters more than its mere presence. Repeated exposure to similar ideas could shrink group differences as participants are most likely to provide familiar responses derived from the previous education especially when responses were taken immediately.

AUTHOR'S RESPONSE:

We agree this is a key gap and have added this to the limitations section: "Although we asked participants whether they had previously received education from a health professional, we did not explore what types of education participants had received. Repeated exposure to similar education may have had an impact on group differences in perceptions as participants who previously received similar information may have had more time to reflect on how this education makes them feel and what treatments they feel are needed."

Minor comments:

Abstract: Page 5 of abstract in the conclusion, the sentence that the participants who received best practice education preferred self -management strategies could be clarified by a percentage or a qualitative descriptor

The phrase "generally similar emotional responses and treatment needs" could be more specific. For example, revise to: "Similar emotional responses across groups, with small

differences in treatment preferences favoring self-management in best practice groups.

AUTHOR'S RESPONSE

Thank you for this suggestion. We have now modified the Conclusion:

Conclusion

"Participants with rotator cuff-related shoulder pain expressed generally similar emotional responses across groups, with small differences in treatment preferences favoring self-management in the best practice education groups compared to those who received structure-focused education. Those in the best practice groups also less frequently reported needing potentially unnecessary treatments (e.g. imaging, injections, and surgery).

Page 9, methods section- Clarify whether the length of the video varied between the groups.

AUTHOR'S RESPONSE

The length of each intervention video was determined by the amount of information provided. For example, the combined *best practice education with pain science messages* video was a longer video as more information was required than the best practice education video alone. We have made this clear in the revised manuscript and acknowledged that different durations could have influenced outcomes.

Methods

"Best practice education (3 minutes, 52 seconds). ...

Best practice education plus pain science messages (5 minutes, 53 seconds). ...

Structure-focused education (2 minutes, 12 seconds). ..."

Discussion

"Given the duration of education varied between groups, we cannot discount that the amount of information may have influenced the results."

Page 19- The term “overmedicalization” in the summary appears without a definition or citation. This term may not be familiar to the readers.

AUTHOR’S RESPONSE

Thank you for noting this. We have replaced the term ‘overmedicalisation’ with ‘unnecessary and/or potentially harmful care’.

Detailed Comments from Reviewer #2:

Reviewer: 2

Dr. WJ Willems, De Lairesse Kliniek, Amsterdam

Comments to the Author:

This study is quite interesting, to evaluate the effect of patient information on patient’s perception.

The both questions are adequate. Given the fact, that most guidelines are rather one sided regarding patient information.

The number of participants is impressive. There are some questions.

AUTHOR’S RESPONSE:

Thank you. We appreciate your time and insightful suggestions for our study.

COMMENT 1:

-Patients were selected from market research panels. They were asked if a diagnosis of the exclusion list was made in the previous period. This might include a bias: patients with e.g. shoulder arthritis or capsulitis could be involved in the study, not (yet) being diagnosed by a health professional.

AUTHOR’S RESPONSE:

Thank you for this feedback. We have added the clarification in the Design section and included this point in the Limitations section. Please see our response to Reviewer 1's **first comment**.

COMMENT 2:

-Looking to the numbers in table 2 and 3, the differences between the 3 types of education in the 30 items (table 2) and 40 items (table 4) are not impressive.
Which statistical program was used for this study?

AUTHOR'S RESPONSE:

Thank you for noting this. Content analyses are typically descriptive studies and are not designed to test statistical hypotheses. Because of this, we did not perform a statistical comparison of themes. We simply presented the percentages which allows readers to make their own interpretation of the importance of the differences. Given we did not perform any statistical inference testing, we used Excel to calculate frequencies and percentages.

COMMENT 3

It is difficult to agree with the conclusions, considering the small differences between the 3 groups.
It is recommended, that the authors explain, why these small differences are significant.

AUTHOR'S RESPONSE:

Thank you for noting this. We have revised our conclusions in this section in response to both reviewers (Reviewer 1's Minor Comments on Page 5) to highlight the small differences between the three groups, pasted below:

Conclusion

"Participants with rotator cuff-related shoulder pain expressed generally similar emotional responses across groups, with small differences in treatment preferences

favoring self-management in the best practice education groups compared to those who received structure-focused education. Those in the best practice groups also less frequently reported needing potentially unnecessary treatments (e.g. imaging, injections, and surgery).

We have labelled the differences as small to temper our conclusions as there is no literature to help us make a judgement about what is an important difference in the codes generated in this study. We also present proportions throughout the paper so readers can decide for themselves whether they see the differences are important or not.

COMMENT 4:

One of the weaknesses of this study is the online presentation of the question. Did the authors consider a (smaller) randomised trial between this online questioning versus a face to face questioning?

AUTHOR'S RESPONSE:

Thank you for this inquiry. Our sample size calculation required 706 participants per group (2118 in total) to detect a 0.5-point difference on the co-primary outcomes of reassurance (quantitative outcomes), assuming a conservative standard deviation of 2.7 (https://jamanetwork.com/journals/jamaneurology/fullarticle/2712902), alpha of 5%, 90% power and 15% drop-out. A smaller trial would not have been sufficient to provide meaningful results for our quantitative outcomes. However, we agree that a future trial embedded in clinical practice would be an important next step for evaluating the effectiveness of different advice and education for shoulder pain.

VERSION 2 - REVIEW

Reviewer 1

Name Vishal, Kavitha

Affiliation Manipal College of Health Professions, Department of Physiotherapy

Date 08-Jan-2026

COI

Dear Authors,

I am happy with the revised version of the manuscript. All my comments have been addressed and this manuscript can be accepted for publication.

VERSION 3 - REVIEW

Reviewer 1

Name Vishal, Kavitha

Affiliation Manipal College of Health Professions, Department of Physiotherapy

Date 20-Jan-2026

COI

Dear Authors,

You have addressed all my concerns in the previous revision.

Thank you
